# Trends in cancer mortality under age 50 in 15 upper-middle and high-income countries

**DOI:** 10.1093/jnci/djae288

**Published:** 2024-11-22

**Authors:** Claudia Santucci, Silvia Mignozzi, Gianfranco Alicandro, Margherita Pizzato, Matteo Malvezzi, Eva Negri, Prabhat Jha, Carlo La Vecchia

**Affiliations:** Department of Clinical Sciences and Community Health, Department of Excellence 2023-2027, University of Milan, 20133 Milan, Italy; Department of Clinical Sciences and Community Health, Department of Excellence 2023-2027, University of Milan, 20133 Milan, Italy; Department of Pathophysiology and Transplantation, University of Milan, 20122 Milan, Italy; Mother and Child Department, Cystic Fibrosis Centre, Fondazione IRCCS Ca’ Granda Ospedale Maggiore Policlinico, 20122 Milan, Italy; Department of Clinical Sciences and Community Health, Department of Excellence 2023-2027, University of Milan, 20133 Milan, Italy; Department of Medicine and Surgery, University of Parma, 43126 Parma, Italy; Department of Medical and Surgical Sciences, University of Bologna, 40138 Bologna, Italy; Centre for Global Health Research, Unity Health Toronto, Dalla Lana School of Public Health, University of Toronto, Toronto, ON M5B 1W8, Canada; Department of Clinical Sciences and Community Health, Department of Excellence 2023-2027, University of Milan, 20133 Milan, Italy

## Abstract

**Background:**

Rising cancer incidence, particularly for colorectal cancer, has been reported in young adults. This study examined whether this is related to an increase in mortality.

**Methods:**

We analyzed World Health Organization mortality data among young adults aged 25-49 years in 15 most populous upper-middle and high-income countries from 1990 to 2021 with reliable data. Midyear populations were retrieved from the United Nations for the American Countries and from the World Health Organization for the other countries. We compared age-standardized mortality rates in 2019-2021 with those in 2009-2011 and performed joinpoint regression analysis for all cancers and selected most common cancer sites: colorectum, pancreas, lung, and breast.

**Results:**

In 2019-2021, the highest age-standardized mortality rates (per 100 000) were in Romanian males (38.6) and Argentinian females (45.9), while the lowest ones were in Japanese males (16.3) and females (22.7). Age-standardized mortality rates for colorectal cancers increased in 2019-2021 compared with 2009-2011 in 9 countries among men and in 7 countries among women. The highest increases were in the United Kingdom (males: +26.1%; females: +33.7%), Canada (males: +25.3%), and Mexico (males: +33.5%; females: +29.7%). Long-term analysis over the last 3 decades showed declining trends in total cancer mortality in the majority of countries, in lung cancer mortality across all countries, and in breast cancer in all countries except in Latin America.

**Conclusions:**

Although mortality from common cancers has generally decreased over the past 3 decades, mortality from colorectal cancer has increased in some countries. This highlights the need to control the obesity epidemic and implement targeted surveillance strategies in young populations.

## Introduction

Overall cancer mortality rates are declining in most high-income countries because of reduced smoking prevalence, particularly in men; advances in infection eradication and food processing methods; improved screening and early diagnosis for common and treatable cancers; and the availability of new treatment options. However, a recent study reported an increase in the global burden of cancer and premature deaths related to malignancies in adults aged 35-64 years and noted that at current rates, most countries are unlikely to meet the Sustainable Development Goal 3.4, which aims to reduce mortality from noncommunicable diseases by one-third.^1^ However, most of these cancer deaths occurred in people aged older than 50 years.

There are growing concerns, including in the media, about increases in cancer diagnoses among young adults. These juvenile cancers are characterized by delayed detection; presentation at an advanced stage, often through nonspecific symptoms; poor differentiation in histology; and rapid progression compared with those diagnosed at later ages. The incidence of early onset cancers—defined as those diagnosed before the age of 50 years—has been increasing over the past decades in the United States and in some European countries for selected neoplasms.^2^ These included mainly colorectal cancer and other neoplasms associated to overweight, such as esophageal and gastric cardia adenocarcinoma, as well as pancreatic, endometrial, and breast cancers. The US Surveillance, Epidemiology, and End Results (SEER) data have documented a 30% increase from 1973 to 2015 in cancer incidence rate among individuals aged 15-39 years.^3^ Age-period-cohort studies also reported higher incidence rates in the younger US generations as compared with the previous ones.^4^ Projections for 2030, based on Global Burden of Disease (GBD) model-based estimates, suggest that the global number of incidence cases and deaths from early onset cancers will increase by 31% and 21%, respectively,^5^ mainly because of population growth. Colorectal, kidney, and thyroid cancers were the main cancers for which a substantial increase has been observed. Data from national and regional European cancer registries also documented increases in colorectal cancer incidence in some countries over the past 2 decades.^6^^,^^7^

Cancer incidence data are affected by changes in diagnostic practices and differences in cancer registration quality and completeness. However, such limitations are less pronounced for mortality data, particularly in younger populations.^8^

We therefore evaluated cancer mortality rates over the last decade and long-term mortality rates from early onset cancers since 1990 in 15 most populous high- and middle-income countries worldwide.

## Methods

We retrieved death counts for young adults aged 25-49 years from the World Health Organization (WHO) database^9^ for all cancers combined and the 17 most common cancer sites, which together accounted for more than 80% of cancer deaths in 2022 among young adults in high-income countries.^10^ These included colorectum, pancreas, lung, breast, oral cavity and pharynx, esophagus, stomach, liver, skin, uterus, ovary, testis, bladder, kidney, Hodgkin lymphoma, non–Hodgkin lymphoma, and leukemias. Because of the relatively low cancer rate at those ages, we chose to include only the most populous countries (≥15 million inhabitants) with high data quality^11^ with at least 500 cancer deaths registered during the last triennium. Additionally, countries with civil registration coverage of cause of death, less than 90% were excluded. From Europe, we included France, Germany, Italy, the Netherlands, Poland, Romania, Spain, and the United Kingdom; from North and South America, we included Canada, the United States, Mexico, Argentina, and Brazil; and we included Japan and Australia from Australasia. Midyear resident population estimates for American countries were extracted from the United Nations Population Division database,^12^ while those for European and Australasian countries were obtained from the WHO database or, when the European data were missing, from the EUROSTAT database.^13^

### Statistical analysis

To assess changes in cancer mortality over the last decade, we compared the age-standardized mortality rates per 100 000 midyear population between the periods 2009-2011 and 2019-2021. The age-standardized mortality rates were calculated for the 25-49 years age group, using the world standard population.^14^ We reported the percent changes in rates between the 2 triennia with the corresponding 95% confidence intervals. Additionally, to evaluate long-term trends, we performed a joinpoint regression analysis^15^ on mortality data from 1990 to the most recent year available for all cancers combined, as well as for most common cancers (colorectal, pancreatic, lung, and breast cancers), which overall account for approximately 50% of total cancer deaths in young adults in the selected countries. Joinpoint analysis for less common cancers was not performed because of the limited number of deaths registered each year in several countries. Joinpoints were identified by iterative testing, ranging from zero to a maximum of 5 joinpoints. Using the final selected model obtained from the joinpoint analysis we also provided the annual percent change for each identified linear segment and the weighted average annual percent change over the entire study period.

Age-standardized mortality rates from all cancers and most common cancers were compared with age-standardized incidence rates obtained from the Global Cancer Observatory of the International Agency for Research on Cancer for the calendar years 2010 and 2017 (Tables S3 and S4).^16^

Statistical analyses were performed using the software R version 4.3.2 (R Development Core Team, 2017), SAS version 9.4 (SAS Institute Inc, Cary, NC, USA), and Joinpoint Regression Program version 5.1.0.0 (Statistical Methodology and Applications Branch, Surveillance Research Program, National Cancer Institute).

This study follows the Guidelines for Accurate and Transparent Health Estimates Reporting for reporting global health estimates.^17^

## Results

**Figure 1.**
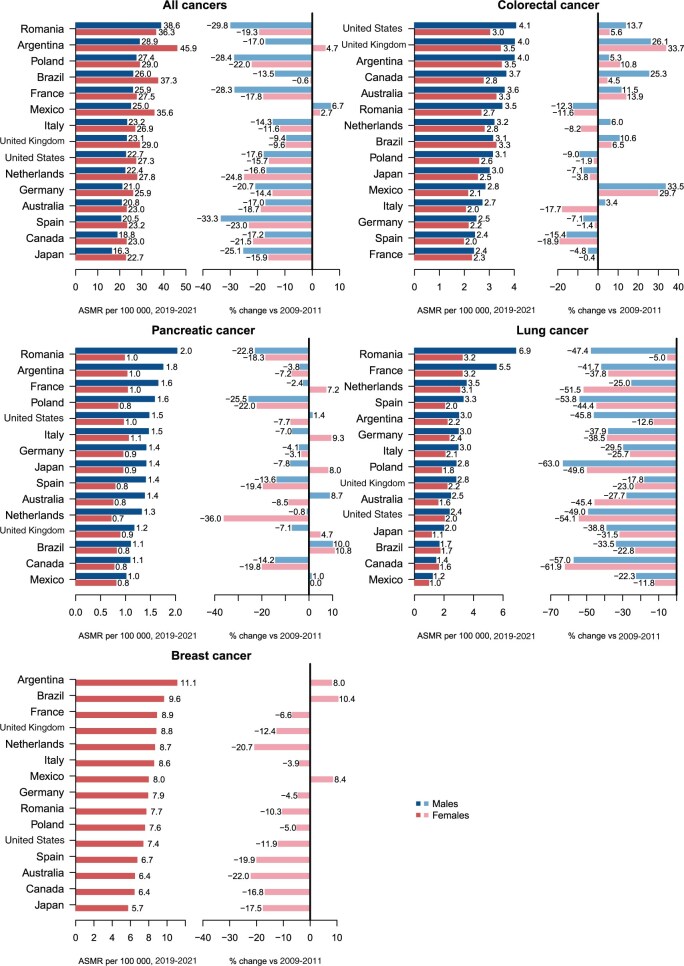
Bar plots reporting the age-standardized (world standard population) mortality rates during 2019-2021 from all cancers, colorectal, pancreatic, lung, and breast cancers with the percent change in rates as compared with 2009-2011 among males and females aged 25-49 years in 15 upper-middle and high-income countries. ASMR = age-standardized mortality rate.

Table 1 and Table 2 give the annual average number of deaths from all cancers and most common cancer sites among males and females aged 25-49 years, registered for the periods 2009-2011 and 2019-2021. These tables also include the age-standardized mortality rates per 100 000 and the percent changes between the 2 periods with the corresponding 95% confidence intervals. Figure 1 shows a ranking of countries based on the age-standardized mortality rate for 2019-2021.

**Table 1.** Age-standardized (world standard population) mortality rates (ASMRs) per 100 000 and annual average number of deaths in parentheses from all cancers, colorectal, pancreatic, and lung cancers among males aged 25-49 years in 15 upper-middle and high-income countries during 2009-2011 and 2019-2021,^a^ with the corresponding percent change in ASMRs between the two periods

Country	All cancers			Colorectum			Pancreas			Lung		
	ASMR 2009-2011	ASMR 2019-2021	%Δ ASMR (95% CI)	ASMR 2009-2011	ASMR 2019-2021	%Δ ASMR (95% CI)	ASMR 2009-2011	ASMR 2019-2021	%Δ ASMR (95% CI)	ASMR 2009-2011	ASMR 2019-2021	%Δ ASMR (95% CI)
	(Deaths)	(Deaths)		(Deaths)	(Deaths)		(Deaths)	(Deaths)		(Deaths)	(Deaths)	
France	36.1 (4049)	25.9 (2899)	−28.3 (−29.2 to −27.4)	2.5 (280)	2.4 (266)	−4.8 (−5.2 to −4.4)	1.7 (193)	1.7 (189)	−2.4 (−2.6 to −2.2)	9.5 (1088)	5.5 (640)	−41.7 (−44.4 to −39.0)
Germany	26.5 (4616)	21.0 (2903)	−20.7 (−21.3 to −20.1)	2.7 (470)	2.5 (343)	−7.1 (−7.6 to −6.6)	1.5 (266)	1.4 (197)	−4.1 (−4.5 to −3.7)	4.8 (881)	3.0 (413	−37.9 (−40.7 to −35.1)
Italy	27.1 (3266)	23.2 (2532)	−14.3 (−14.8 to −13.8)	2.6 (323)	2.7 (301)	3.4 (3.0 to 3.8)	1.6 (195)	1.5 (169)	−7.0 (−7.8 to −6.2)	4.2 (521)	3.0 (342)	−29.5 (−32.0 to −27.0)
Netherlands	26.8 (880)	22.4 (647)	−16.6 (−17.5 to −15.7)	3.0 (101)	3.2 (93)	6.0 (4.9 to 7.1)	1.3 (45)	1.3 (39)	−0.8 (−0.7 to −0.9)	4.7 (159)	3.5 (103)	−25.0 (−28.4 to −21.6)
Poland	38.3 (2560)	27.4 (2004)	−28.4 (−29.3 to −27.5)	3.4 (230)	3.1 (230)	−9.0 (−9.8 to −8.2)	2.1 (140)	1.6 (116)	−25.5 (−29.1 to −21.9)	7.6 (503)	2.8 (206)	−63.0 (−68.7 to −57.3)
Romania	55.0 (2065)	38.6 (1534)	−29.8 (−31.4 to −28.2)	4.0 (153)	3.5 (142)	−12.3 (−14.5 to −10.1)	2.6 (97)	2.0 (83)	−22.8 (−28.2 to −17.4)	13.1 (476)	6.9 (277)	−47.4 (−53.4 to −41.4)
Spain	30.8 (2939)	20.5 (1986)	−33.3 (−34.4 to −32.2)	2.9 (272)	2.4 (239)	−15.4 (−16.9 to −13.9)	1.6 (155)	1.4 (143)	−13.6 (−15.3 to −11.9)	7.1 (679)	3.3 (335)	−53.8 (−57.7 to −49.9)
United Kingdom	25.5 (2950)	23.1 (2587)	−9.4 (−9.7 to −9.1)	3.2 (369)	4.0 (450)	26.1 (23.7 to 28.5)	1.3 (150)	1.2 (132)	−7.1 (−8.0 to −6.2)	3.4 (407)	2.8 (317)	−17.8 (−19.4 to −16.2)
Canada	22.7 (1546)	18.8 (1216)	−17.2 (−18.0 to −16.4)	2.9 (201)	3.7 (239)	25.3 (22.1 to 28.5)	1.3 (89)	1.1 (70)	−14.2 (−16.9 to −11.5)	3.4 (241)	1.5 (94)	−57.0 (−66.4 to −47.6)
United States	27.5 (15 589)	22.7 (12332)	−17.6 (−17.9 to −17.3)	3.6 (2035)	4.1 (2208)	13.7 (14.3 to 13.1)	1.5 (837)	1.5 (796)	1.4 (1.2 to 1.6)	4.6 (2707)	2.4 (1273)	−49.0 (−51.2 to −46.8)
Argentina	34.8 (2175)	28.9 (2157)	−17.0 (−17.6 to −16.4)	3.8 (239)	4.0 (300)	5.3 (4.6 to 6.0)	1.8 (110)	1.8 (127)	−3.8 (−4.3 to −3.3)	5.5 (332)	3.0 (218)	−45.8 (−50.8 to −40.8)
Brazil	30.0 (10 326)	26.0 (10 096)	−13.5 (−13.7 to −13.3)	2.8 (980)	3.1 (1228)	10.6 (10.0 to 11.2)	1.0 (338)	1.1 (422)	10.0 (9.0 to 11.0)	2.5 (846)	1.7 (642)	−33.5 (−35.8 to −31.2)
Mexico	23.4 (4274)	25.0 (5382)	6.7 (6.5 to 6.9)	2.1 (385)	2.8 (604)	33.5 (30.8 to 36.2)	1.0 (175)	1.0 (213)	1.0 (0.7 to 1.3)	1.6 (280)	1.2 (262)	−22.3 (−24.5 to −20.1)
Japan	21.8 (4789)	16.3 (3683)	−25.1 (−25.7 to −24.5)	3.2 (717)	3.0 (695)	−7.1 (−7.5 to −6.7)	1.5 (338)	1.4 (335)	−7.8 (−8.4 to −7.2)	3.3 (721)	2.0 (477)	−38.8 (−41.3 to −36.3)
Australia	25.1 (1024)	20.8 (925)	−17.0 (−17.8 to −16.2)	3.2 (132)	3.6 (162)	11.5 (9.9 to 13.1)	1.3 (52)	1.4 (61)	8.7 (6.6 to 10.8)	3.4 (140)	2.5 (109)	−27.7 (−31.7 to −23.7)

a 2021 data were not available for France, Germany, Italy, Romania, the UK, Canada, the USA, Argentina, Brazil, and Mexico. For these countries the ASMRs refer to the biennium 2019-2020. ASMR = age-standardized mortality rate using the world standard population; CI = confidence interval.

**Table 2.** Age-standardized (world standard population) mortality rates (ASMRs) per 100 000 and the annual average number of deaths in parentheses from all cancers, colorectal, pancreatic, lung, and breast cancers among females aged 25-49 years in 15 upper-middle and high-income countries during 2009-2011 and 2019-2021,^a^ with the corresponding percent change in ASMRs between the two periods

Country	All cancers			Colorectum			Pancreas			Lung			Breast		
	ASMR 2009-2011	ASMR 2019-2021	%Δ ASMR (95% CI)	ASMR 2009-2011	ASMR 2019-2021	%Δ ASMR (95% CI)	ASMR 2009-2011	ASMR 2019-2021	%Δ ASMR (95% CI)	ASMR 2009-2011	ASMR 2019-2021	%Δ ASMR (95% CI)	ASMR 2009-2011	ASMR 2019-2021	%Δ ASMR (95% CI)
	(Deaths)	(Deaths)		(Deaths)	(Deaths)		(Deaths)	(Deaths)		(Deaths)	(Deaths)		(Deaths)	(Deaths)	
France	33.5 (3839)	27.5 (3180)	−17.8 (−18.3 to −17.3)	2.3 (264)	2.3 (265)	−0.4 (−0.3 to −0.5)	1.0 (113)	1.0 (123)	7.2 (5.8 to 8.6)	5.2 (612)	3.2 (383)	−38.0 (−40.9 to −34.7)	9.5 (1099)	8.9 (1030)	−6.6 (−6.9 to −6.3)
Germany	30.3 (5060)	25.9 (3517)	−14.4 (−14.8 to −14.0)	2.2 (370)	2.2 (294)	−1.4 (−1.4 to −1.4)	1.0 (170)	1.0 (131)	−3.1 (−3.4 to −2.8)	3.8 (671)	2.4 (323)	−39.0 (−41.8 to −35.2)	8.3 (1396)	7.9 (1075)	−4.5 (−4.7 to −4.3)
Italy	30.4 (3740)	26.9 (2991)	−11.6 (−11.9 to −11.3)	2.5 (310)	2.1 (234)	−18.0 (−19.6 to −15.8)	1.0 (122)	1.1 (123)	9.3 (7.6 to 11.0)	2.8 (352)	2.1 (245)	−26.0 (−28.5 to −22.9)	8.9 (1114)	8.6 (967)	−3.9 (−4.1 to −3.7)
Netherlands	36.9 (1201)	27.8 (804)	−24.8 (−26.0 to −23.6)	3.1 (100)	2.8 (82)	−8.2 (−9.4 to −7.0)	1.1 (37)	0.7 (21)	−36.0 (−47.3 to −24.7)	6.3 (213)	3.1 (91)	−52.0 (−58.8 to −44.2)	10.9 (360)	8.7 (251)	−20.7 (−22.5 to −18.9)
Poland	37.2 (2475)	29.0 (2105)	−22.0 (−22.7 to −21.3)	2.6 (175)	2.6 (188)	−1.9 (−2.0 to −1.8)	1.1 (72)	0.9 (61)	−22.0 (−26.0 to −18.0)	3.7 (241)	1.8 (133)	−50.0 (−55.5 to −43.7)	8.0 (527)	7.6 (557)	−5.0 (−5.3 to −4.7)
Romania	45.0 (1736)	36.3 (1355)	−19.3 (−20.4 to −18.2)	3.0 (117)	2.7 (101)	−12.0 (−14.0 to −9.2)	1.2 (46)	1.0 (37)	−18.3 (−24.6 to −12.0)	3.4 (129)	3.2 (124)	−5.0 (−5.8 to −4.2)	8.6 (330)	7.7 (294)	−10.3 (−11.5 to −9.1)
Spain	30.1 (2840)	23.2 (2221)	−23.0 (−23.7 to −22.3)	2.4 (228)	2.0 (189)	−19.0 (−20.8 to −17.0)	1.0 (92)	0.8 (79)	−19.4 (−22.6 to −16.2)	3.7 (348)	2.1 (205)	−44.0 (−48.6 to −40.2)	8.4 (801)	6.7 (659)	−19.9 (−21.0 to −18.8)
United Kingdom	32.1 (3804)	29 (3320)	−9.6 (−9.9 to −9.3)	2.6 (305)	3.5 (395)	33.7 (37.1 to 30.3)	0.9 (104)	0.9 (103)	4.7 (3.7 to 5.7)	2.9 (350)	2.2 (255)	−23.0 (−25.4 to −20.6)	10 (1210)	8.8 (1011)	−12.4 (−13.0 to −11.8)
Canada	29.3 (1975)	23 (1496)	−21.5 (−22.4 to −20.6)	2.6 (179)	2.8 (180)	4.5 (3.8 to 5.2)	1.0 (66)	0.8 (50)	−19.8 (−24.5 to −15.1)	4.3 (303)	1.6 (106)	−62.0 (−70.8 to −53.0)	7.7 (520)	6.4 (420)	−16.8 (−18.1 to −15.5)
United States	32.4 (18 222)	27.3 (14 882)	−15.7 (−15.9 to −15.5)	2.9 (1618)	3.0 (1645)	5.6 (5.3 to 5.9)	1.0 (599)	1.0 (521)	−7.7 (−8.2 to −7.2)	4.4 (2562)	2.0 (1098)	−54.0 (−56.5 to −51.7)	8.4 (4734)	7.4 (4029)	−11.9 (−12.2 to −11.6)
Argentina	43.9 (2930)	45.9 (3595)	4.7 (4.5 to 4.9)	3.2 (209)	3.5 (274)	10.8 (9.4 to 12.2)	1.1 (72)	1.0 (81)	−7.2 (−8.5 to −5.9)	2.5 (165)	2.2 (171)	−13.0 (−14.2 to −11.0)	10.3 (681)	11.1 (865)	8.0 (7.4 to 8.6)
Brazil	37.5 (13 587)	37.3 (15 175)	−0.6 (−0.6 to −0.6)	3.1 (1109)	3.3 (1326)	6.5 (6.1 to 6.9)	0.7 (264)	0.8 (327)	10.8 (9.5 to 12.1)	2.2 (796)	1.7 (688)	−23.0 (−24.4 to −21.2)	8.7 (3157)	9.6 (3947)	10.4 (10.1 to 10.7)
Mexico	34.7 (6839)	35.6 (8384)	2.7 (2.6 to 2.8)	1.7 (325)	2.1 (506)	29.7 (27.0 to 32.4)	0.8 (155)	0.8 (188)	0 (−0.1 to 0.1)	1.1 (215)	1.0 (227)	−12.0 (−13.1 to −10.5)	7.3 (1443)	8.0 (1874)	8.4 (8.0 to 8.8)
Japan	26.9 (5849)	22.7 (4999)	−15.9 (−16.2 to −15.6)	2.6 (573)	2.5 (561)	−3.8 (−4.0 to −3.6)	0.9 (191)	1.0 (218)	8.0 (6.9 to 9.1)	1.7 (368)	1.2 (264)	−32.0 (−34.2 to −28.8)	6.9 (1513)	5.7 (1285)	−17.5 (−18.2 to −16.8)
Australia	28.3 (1175)	23.0 (1047)	−18.7 (−19.6 to −17.8)	2.9 (119)	3.3 (150)	13.9 (11.9 to 15.9)	0.8 (34)	0.8 (34)	−8.5 (−10.5 to −6.5)	2.9 (123)	1.6 (73)	−45.0 (−52.9 to −37.9)	8.3 (346)	6.5 (294)	−22.0 (−23.9 to −20.1)

a 2021 data were not available for France, Germany, Italy, Romania, the UK, Canada, the USA, Argentina, Brazil, and Mexico. For these countries the ASMRs refer to the biennium 2019-2020. ASMR = age-standardized mortality rate using the world standard population; CI = confidence interval.

**Figure 2.**
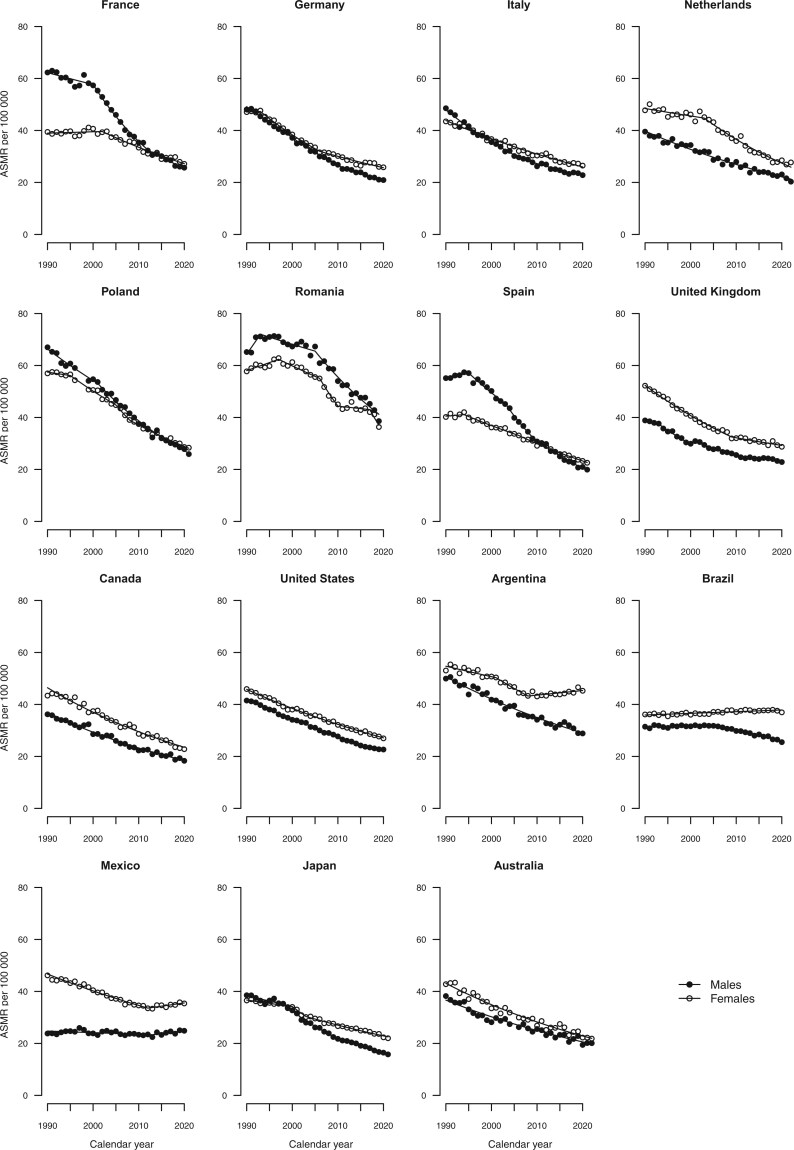
Joinpoint analysis of age-standardized (world standard population) mortality rates from all cancers combined per 100 000 population aged 25-49 years in 15 upper-middle and high-income countries by sex. ASMR = age-standardized mortality rate.

**Figure 3.**
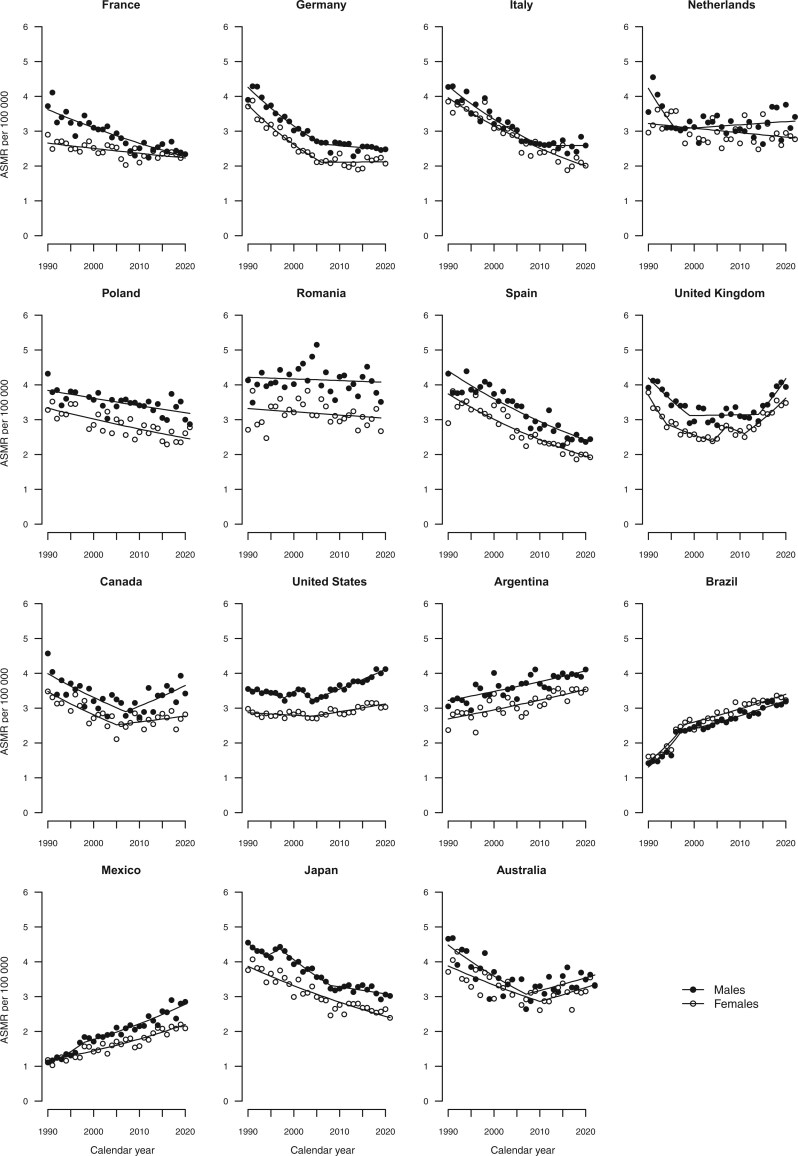
Joinpoint analysis of age-standardized (world standard population) mortality rates from colorectal cancer per 100 000 population aged 25-49 years in 15 upper-middle and high-income countries by sex. ASMR = age-standardized mortality rate.

**Figure 4.**
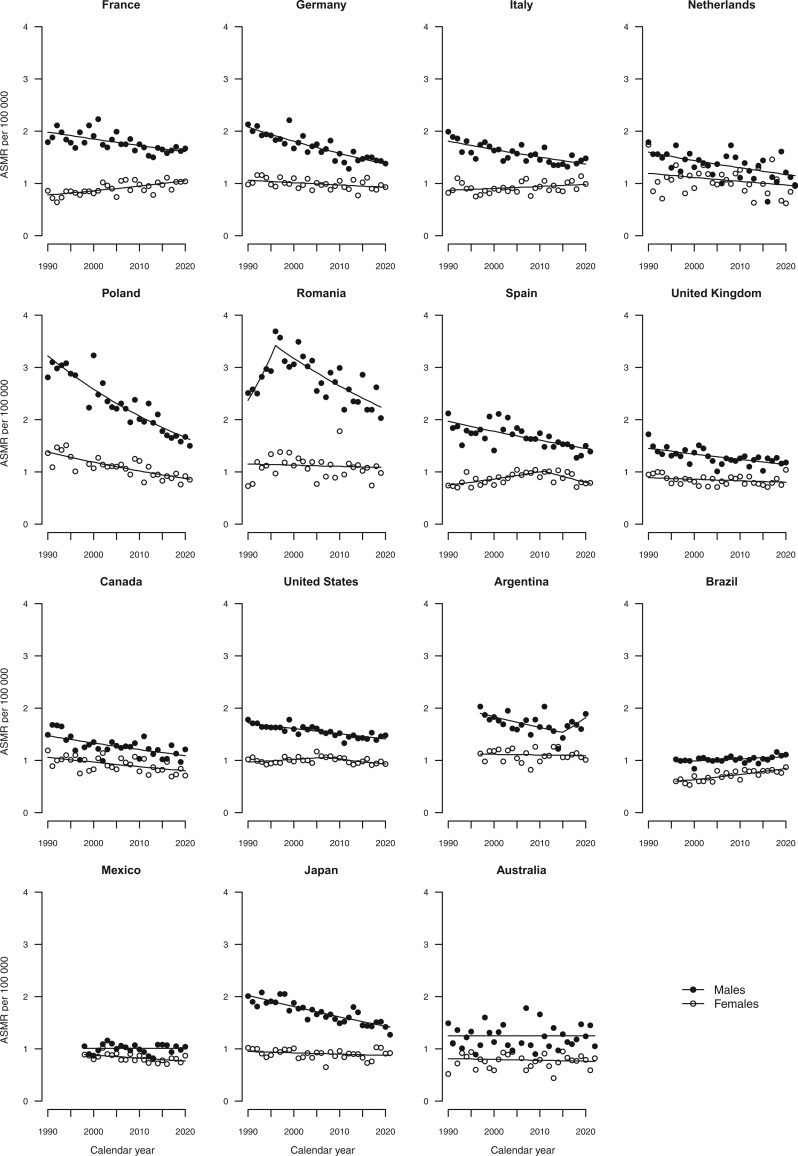
Joinpoint analysis of age-standardized (world standard population) mortality rates from pancreatic cancer per 100 000 population aged 25-49 years in 15 upper-middle and high-income countries by sex. ASMR = age-standardized mortality rate.

**Figure 5.**
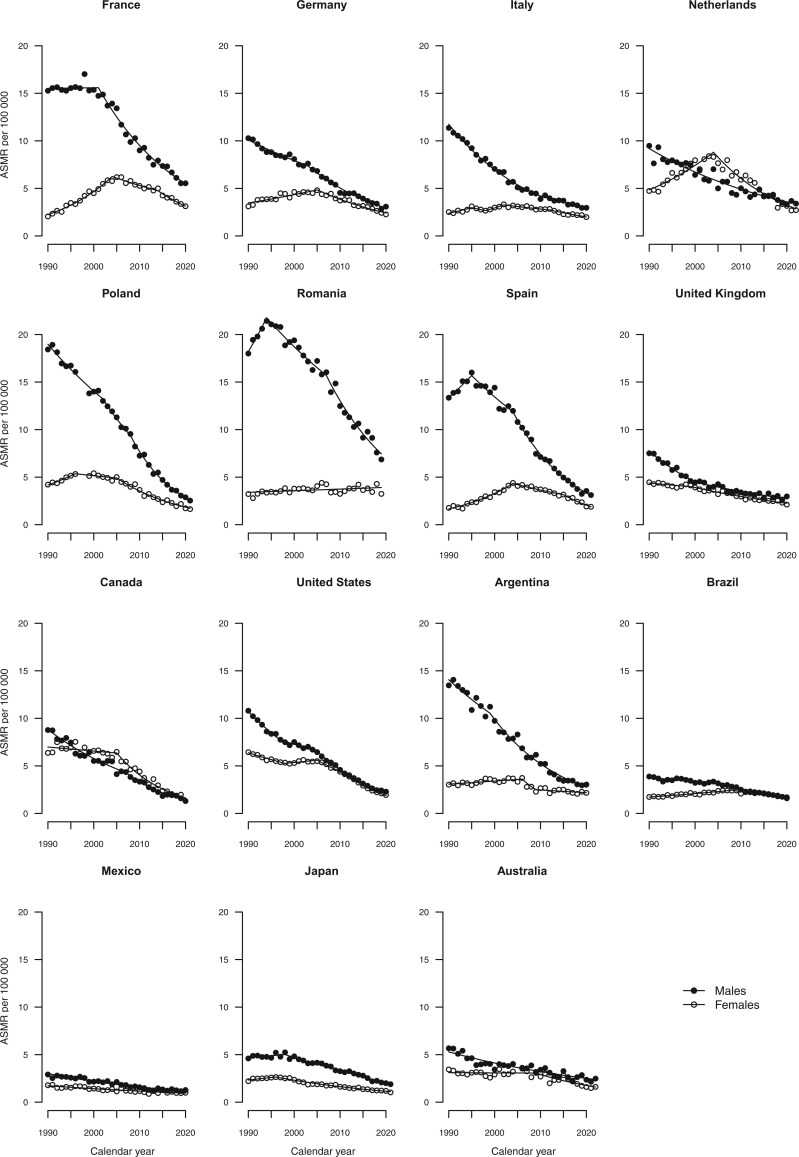
Joinpoint analysis of age-standardized (world standard population) mortality rates from lung cancer per 100 000 population aged 25-49 years in 15 upper-middle and high-income countries by sex. ASMR = age-standardized mortality rate.

**Figure 6.**
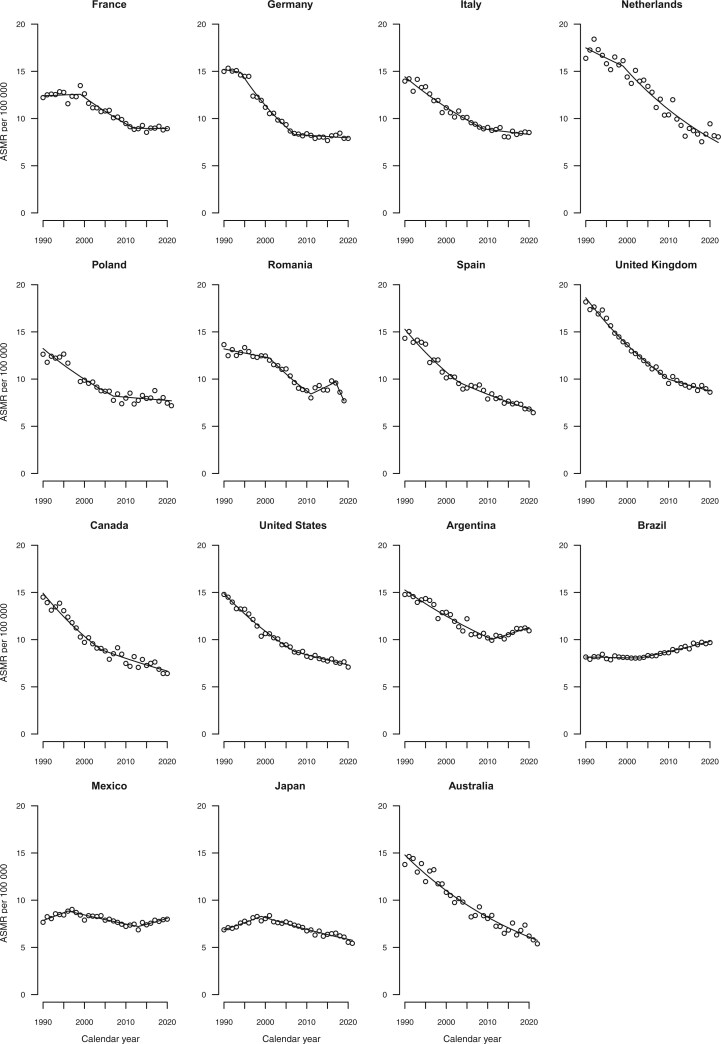
Joinpoint analysis of age-standardized (world standard population) mortality rates from breast cancers per 100 000 females aged 25-49 years in 15 upper-middle and high-income countries. ASMR = age-standardized mortality rate.

Figures 2-6 give the trends in age-standardized mortality rates from all cancers combined, colorectal, pancreatic, lung, and breast cancers, along with the fitted lines obtained from the joinpoint models. Annual percent changes are reported in Tables S1 and S2.

### All cancers

In 2019-2021 among males, Romania showed the highest age-standardized mortality rate (38.6/100 000), while Japan had the lowest rate (16.3; Tables 1 and 2 and Figure 1). Male age-standardized mortality rates were lower in 2019-2021 than in 2009-2011 in all countries except Mexico (+6.7%). In 2019-2021, female age-standardized mortality rates ranged from 22.7 in Japan to 45.9 in Argentina. Age-standardized mortality rates were lower in the most recent triennium as compared with 2009-2011 in all countries with the exception of Argentina (+4.7%) and Mexico (+2.7%).

Over the period 1990-2021, the age-standardized mortality rates declined across all countries considered and both sexes, except for Mexico and Argentina for females, where the declining trends halted in the mid-2000s, and in Mexican and Brazilian females, whose mortality remained stable (Figure 2).

### Colorectal cancer

In 2019-2021, age-standardized mortality rates per 100 000 from colorectal cancer ranged from 2.4 in France and Spain to 4.1 in the United States for males and from 2.0 in Spain to 3.5 in the United Kingdom for females (Tables 1 and 2 and Figure 1). Compared with the period 2009-2011, age-standardized mortality rates in 2019-2021 increased in Italy (+3.4% in males), the Netherlands (+6.0% in males), the United Kingdom (+26.1% in males and +33.7% in females), Canada (+25.3% in males and +4.5% in females), the United States (+13.7% in males and +5.6% in females), Argentina (+5.3% in males and +10.8% in females), Brazil (+10.6% in males and +6.5% in females), Mexico (+33.5% in males and +29.7% in females), and Australia (+11.5% in males and+13.9% in females).

Over the last 3 decades, trends in colorectal cancer mortality in young adults have been declining in France, Poland, Spain, and Japan (Figure 3). Conversely, upward trends were observed in the United Kingdom, Canada, the United States, and Australia, where death rates have appreciably increased since the mid-2000s for both sexes. In Mexico, Argentina, and Brazil, age-standardized mortality rates have increased over the entire period. In Germany and among men in Italy, the declining trends observed in the 1990s and early 2000s have leveled off. In the Netherlands, rates have remained stable since 1990.

### Pancreatic cancer

In 2019-2021, age-standardized mortality rates per 100 000 from pancreatic cancer ranged from 1.0/100 000 in Mexico to 2.0 deaths in Romania for males, whereas rates for females were almost similar in all countries, with values less than or equal to 1.0 (Tables 1 and 2 and Figure 1). Higher mortality rates were observed in 2019-2021 than in 2009-2011 in France (+7.2% in females), Italy (+9.3% in females), the United Kingdom (+4.7% in females), the United States (+1.4% in males), Brazil (+10.0% in males and +10.8% in females), Mexico (+1.0% in males), Japan (+8.0% in females), and Australia (+8.7% in males).

Pancreatic cancer mortality trends between 1990 and 2021 were favorable or stable for both sexes in most countries, except for some upward trends in Brazil among both sexes, in France and Italy among females, and in Argentina in men in the last quinquennium (Figure 4).

### Lung cancer

In 2019-2021, the highest age-standardized mortality rate per 100 000 from lung cancer in males was in Romania (6.9), and the lowest one was in Mexico (1.2; Tables 1 and 2 and Figure 1). Among females, the highest rates in 2019-2021 were in Romania and France (3.2), and the lowest was in Mexico (1.0). Compared with 2009-2011, substantial decreases in age-standardized mortality rates were observed in both sexes and all countries considered.

All selected countries showed a decreasing trend in lung cancer mortality over the period 1990-2021 (Figure 5), with more pronounced decreases observed in males. The only exception was among Romanian females, who showed a slight upward trend over the entire period considered (Table S2).

### Breast cancer

In 2019-2021, age-standardized mortality rates per 100 000 from breast cancer varied from 5.7 in Japan to 11.1 in Argentina (Tables 1 and 2 and Figure 1). Compared with the period 2009-2011, all countries considered showed lower mortality rates, except for Argentina (+8.0%), Brazil (+10.4%), and Mexico (+8.4%).

Over the period 1990-2021, the age-standardized mortality rates appreciably declined in all selected countries with the exception of Latin American countries (Figure 6). In Argentina and Mexico, mortality rates started to increase since around 2010, whereas in Brazil since around 2000.

### Other cancer sites

Tables S5 and S6 give the annual average number of deaths and age-standardized mortality rates per 100 000 in selected countries from oral cavity and pharynx, esophageal, stomach, liver, skin, testis, bladder, kidney cancers, Hodgkin lymphoma, non–Hodgkin lymphoma and leukemias, respectively, among males and females aged 25-49 years for the periods 2009-2011 and 2019-2021, along with the corresponding percent changes. Compared with the period 2009-2011, most of the selected countries showed favorable trends in mortality rates.

## Discussion

Our study, which includes major upper-middle and high-income countries worldwide, found declining trends in mortality from total cancers and several cancer sites in young adults over the last 3 decades. However, we also documented increased trends for colorectal cancer mortality rates in 2019-2021 compared with 2009-2011 in 9 countries among men (Italy, the Netherlands, the United Kingdom, Canada, the United States, Argentina, Brazil, Mexico, and Australia) and in 7 countries among women (the United Kingdom, Canada, the United States, Argentina, Brazil, Mexico, and Australia). Additionally, higher mortality rates from pancreatic cancer in 2019-2021 were noted in 4 countries among men (the United States, Brazil, Mexico, and Australia) and in 5 countries among women (France, Italy, the United Kingdom, Brazil and Japan).

Our results on mortality contrast with reports of rising cancer incidence in young adults in most of the countries included in our analysis.^2-5^^,^^16^ Caution is required when interpreting incidence trends because of general improvements in registration.^8^ The incidence of selected neoplasms—such as breast, thyroid, and to a lesser extent, lung, colorectum, prostate, and uterus—is influenced by earlier and improved diagnosis, even in those aged younger than 50 years and in the absence of organized screening programs. Additionally, lifestyle factors associated with obesity and diabetes likely contributed to the rising incidence of colorectal and pancreatic cancers in young adults thereby affecting mortality trends in some countries.^18^^,^^19^

Globally, colorectal cancer is the second leading cause of cancer death and the leading cause among nonsmokers of both sexes.^10^ Whereas there have been persistent declines in mortality across all ages and both sexes in Europe and the United States, recent upward trends have been observed among young adults.^20^^,^^21^ In the United States, among those younger than 50 years, colorectal cancer is the leading cause of cancer death among men and the second leading among women.^22^

A family history of colorectal cancer and adenomatous polyps, as well as a diagnosis of inflammatory bowel disease, are predisposing factors for developing colorectal cancer, particularly among young adults.^23^

Nonetheless, the major drivers of the recent upward trends are likely the unfavorable changes in some known risk factors (obesity, sedentariness, and western dietary patterns), at times not counterbalanced by access to early detection services.

Overweight and obesity are key risk factors,^24^ with excess body weight contributing to approximately 30% of new cases in the United States from 1992 to 2016.^25^ Given the global increase in overweight and obesity prevalence across all ages and both sexes since 1980,^26^ these factors are likely the major drivers of the recent upward trends.^27^^,^^28^ In the 20-39 years age group, in both sexes, countries like the United Kingdom, Canada, the United States, and Mexico, where increases in colorectal cancer mortality have been observed, also showed substantial rises in the prevalence of obesity in the young when comparing data from 1990 to 2022.^29^ Alcohol consumption, diabetes, a sedentary lifestyle, and a Western-style diet have also been related to early onset colorectal cancer.^25^^,^^28^^,^^30^

The disparity in mortality trends among younger groups compared with older groups has been mainly attributed to the absence of organized screening programs in individuals aged younger than 50 years. Additionally, younger patients may present with nonspecific colorectal symptoms, leading to delays in diagnosis, even in high-income countries, and tend to develop more aggressive histotypes characterized by rapid progression.^31^ In fact, a higher percentage of patients younger than 50 years are diagnosed with advanced cancer than older patients, probably because of the lack of screening and the prevalence of nonspecific symptoms in this age group.^31^^,^^32^ A study from the Stanford Cancer Institute found that 72% of young-onset colorectal cancer cases (diagnosed before age 50 years) were at an advanced stage compared with 63% of those diagnosed in individuals aged older than 50 years.^31^

We observed some upward trends in mortality from pancreatic cancer, which have also been detected in incidence data for countries such as France and the United Kingdom among females, but not in Japan among females or in Australia among males (Tables S3 and S4).^2^^,^^33^^,^^34^ Consistent with our results, a study from the United States found that mortality from pancreatic cancer remained stable over the last 2 decades among young adults.^35^ Despite reductions in smoking rates in high-income countries, increasing trends in the prevalence of obesity, diabetes, and alcohol consumption may have contributed to the unfavorable incidence rates.^36^^,^^37^ Younger patients are more likely to receive treatment than their older counterparts.^38^ However, because of its limited response to therapy and rapid progression, the prognosis of pancreatic cancer remains poor, with less than 10% of patients surviving beyond 5 years.^36^^,^^39^ Thus, trends in mortality consistently reflect those in incidence.

Lung cancer mortality has declined in most countries,^16^ but rates in Central and Eastern European countries have remained higher compared with other countries considered for both sexes.^40^ Smoking control policies are reflected in lung cancer mortality trends starting from young adults, which therefore represent an important indication for future trends in middle and elderly population in the absence of widespread smoking cessation.^8^ The decline in mortality among women was not as pronounced as that for men, likely reflecting the different patterns of cessation, with smoking prevalence in women peaking later than in men.^41^ Romanian women did not show a decrease in rates, and Polish women reported persistently high mortality from lung cancer, though leveling off over recent years.

Breast cancer is the leading cause of cancer deaths among women aged younger than 50 years, accounting for approximately 25% of overall cancer deaths in this age group. Over the past decade, most countries showed favorable trends in breast cancer mortality, with the exceptions of Mexico, Brazil, and Argentina. Delayed diagnosis, together with limited access to innovative treatments, have an impact on breast cancer mortality.^42^ Moreover, younger women more often develop aggressive breast cancers, which are associated with a higher risk of recurrence and death.^43^ Risk factors for breast cancer include early menarche, late first pregnancy, nulliparity, and absence of breastfeeding.^44^ No appreciable improvement was observed in those risk factors in the countries considered over the last decades. Besides, changes in reproductive factors, physical inactivity, alcohol consumption, and Westernization of diet may have played a role in the rising incidence of premenopausal breast cancer observed in selected countries during the last decades.^2^^,^^45^ However, early diagnosis has likely contributed to increased incidence and decreased mortality from breast cancer among young women.^46^^,^^47^ According to the International Agency for Research on Cancer estimates (Tables S3 and S4), breast cancer incidence has shown a stable or slightly increasing trend worldwide over the last decade. This divergence between mortality and incidence trends is likely due to effective treatment and improved disease management.^48-50^

Compared with cancer patients diagnosed at older ages, survivors diagnosed at a young age face additional health problems later in life such as infertility, cardiovascular disease, and cancer recurrence.^51-53^ Moreover, a diagnosis of a juvenile tumor could prevent or limit the possibility of obtaining loans or insurance in a market-oriented health-care system. Therefore, despite representing a minority of all cancer cases, the social and health burden of a diagnosis and cancer deaths at ages younger than 50 years are of particular relevance.

Our study provides a comprehensive and updated analysis of patterns and temporal trends in cancer mortality in young adults, filling a gap in existing research. The analysis is based on the most updated and reliable official mortality data provided to the WHO by national statistics offices. To enhance the robustness of our analyses, we restricted our study to major countries with high data coverage and good data quality.

The main limitation of this study is the exclusion of certain countries and regions, such as China, India, other Asian countries, African countries and some South American countries, because of low data availability and/or quality.^54^ Additionally, the low number of deaths from less common cancers made it difficult to precisely estimate their mortality changes over time.

In conclusion, overall cancer mortality in young adults decreased over the last 3 decades. The reduction in mortality from the most common cancers in both sexes likely reflects advancements in cancer prevention (mainly tobacco control), along with improvements in diagnosis and treatment. Colorectal cancer mortality in the young, however, has increased in several middle- and high-income countries. This highlights the need for surveillance strategies targeted to high-risk individuals, such as those with a family history of colorectal cancer and obese patients, as well as the promotion of healthy lifestyles, including body weight control, and equitable access to effective therapies.

## Supplementary Material

djae288_Supplementary_Data

## Data Availability

Data derived from a source in the public domain. We retrieved death counts from the World Health Organization (WHO) database and resident population data from the WHO database, the United Nations Population Division database, and EUROSTAT.
