# Supplementary material for: Trends in cancer mortality under age 50 in 15 upper-middle and high-income countries
Source: J Natl Cancer Inst. 2024 Nov 22;117(4):747–60. doi: 10.1093/jnci/djae288 (PMC11972687; doi:10.1093/jnci/djae288)
Supplement: djae288_Supplementary_Data [file djae288_supplementary_data.pdf]

SUPPLEMENTARY MATERIAL

Manuscript title: Trends in cancer mortality under age 50 in 15 upper-middle and high-income countries

Table of contents

Supplementary Table 1. Joinpoint analysis from 1990 to the most available year for males aged 25-49 years, by country and cancer site. 2

Supplementary Table 2. Joinpoint analysis from 1990 to the most available year for females aged 25-49 years, by country and cancer site. 4

Supplementary Table 3. Age-adjusted incidence rates per 1000,000 males from colorectal, pancreatic, lung cancer and all cancers combined among males aged 25-49 years in major countries worldwide in selected countries worldwide in 2010 and 2017. 7

Supplementary Table 4. Age-adjusted incidence rates per 1000,000 females from colorectal, pancreatic, lung cancer and all cancers combined among males aged 25-49 years in major countries worldwide in selected countries worldwide in 2010 and 2017. 8

Supplementary Table 5. Age-standardized (world population) mortality rates per 100,000 males aged 25-49 years for selected cancer sites in major countries worldwide in 2009-2011 and 2019-2021°, with the corresponding percent change between the two calendar periods. 9

Supplementary Table 6. Age-standardized (world population) mortality rates per 100,000 females aged 25-49 years for selected cancer sites in major countries worldwide in 2009-2011 and 2019-2021°, with the corresponding percent change between the two calendar periods. 13

Supplementary Table 1. Joinpoint analysis from 1990 to the most available year for males aged 25-49 years, by country and cancer site.

	Years1	APC1	Years2	APC2	Years3	APC3	Years4	APC4	Years5	APC5	AAPC
**All cancers**											
France	1990-2000	-0.8*	2000-2012	-4.7*	2012-2020	-3*					-2.9*
Germany	1990-1999	-2.7*	1999-2011	-3.3*	2011-2020	-2.3*					-2.8*
Italy	1990-2007	-2.9*	2007-2020	-1.9*							-2.5*
Netherlands	1990-2022	-1.9*									-1.9*
Poland	1990-2001	-2*	2001-2021	-3.5*							-2.9*
Romania	1990-1993	3.8*	1993-2005	-0.8*	2005-2019	-3.3*					-1.5*
Spain	1990-1995	0.7	1995-2003	-2.8*	2003-2009	-5.5*	2009-2021	-4.1*			-3.3*
UK	1990-1993	-1.3	1993-1999	-3.3*	1999-2002	-0.6	2002-2011	-2.1*	2011-2020	-0.7*	-1.7*
Canada	1990-2020	-2.3*									-2.3*
USA	1990-2003	-2*	2003-2016	-2.4*	2016-2020	-1.3*					-2*
Mexico	1990-2013	-0.3*	2013-2020	0.9*							0
Argentina	1990-2020	-1.8*									-1.8*
Brazil	1990-2006	0.1	2006-2020	-1.4*							-0.6*
Japan	1990-1998	-1.1*	1998-2009	-3.9*	2009-2021	-2.9*					-2.8*
Australia	1990-2022	-1.9*									-1.9*
**Colorectum**											
France	1990-2020	-1.5*									-1.5*
Germany	1990-2006	-3*	2006-2020	-0.5							-1.8*
Italy	1990-2010	-2.5*	2010-2020	0							-1.7*
Netherlands	1990-1996	-5.3*	1996-2022	0.3							-0.8
Poland	1990-2021	-0.6*									-0.6*
Romania	1990-2019	-0.1									-0.1
Spain	1990-2021	-2*									-2*
UK	1990-1999	-3.3*	1999-2014	0.1	2014-2020	4.8*					0
Canada	1990-2007	-1.9*	2007-2020	1.8*							-0.3
USA	1990-1998	-1*	1998-2001	2.5	2001-2004	-3	2004-2020	1.5*			0.5
Mexico	1990-1998	5.9*	1998-2020	2.3*							3.2*
Argentina	1990-2020	0.8*									0.8*
Brazil	1990-1997	8.5*	1997-2020	1.5*							3.1*
Japan	1990-1994	-2.1	1994-1997	1.7	1997-2008	-2.5*	2008-2021	-0.6*			-1.3*
Australia	1990-2007	-2.2*	2007-2022	1.1*							-0.7*
**Pancreas**											
France	1990-2020	-0.7*									-0.7*

	Years1	APC1	Years2	APC2	Years3	APC3	Years4	APC4	Years5	APC5	AAPC
Germany	1990-2020	-1.4*									-1.4*
Italy	1990-2020	-0.9*									-0.9*
Netherlands	1990-2022	-1*									-1*
Poland	1990-2021	-2.2*									-2.2*
Romania	1990-1996	6.3*	1996-2019	-1.8*							-0.2
Spain	1990-2021	-1*									-1*
UK	1990-2020	-0.8*									-0.8*
Canada	1990-2020	-1*									-1*
USA	1990-2020	-0.7*									-0.7*
Mexico	1998-2020	0									0
Argentina	1997-2015	-1.2*	2015-2020	3.4							-0.2
Brazil	1996-2020	0.4*									0.4*
Japan	1990-2021	-1.1*									-1.1*
Australia	1990-2022	0									0
**Lung**											
France	1990-2001	0	2001-2020	-5.3*							-3.4*
Germany	1990-2003	-2.4*	2003-2020	-5.3*							-4*
Italy	1990-2009	-5.1*	2009-2020	-3.4*							-4.5*
Netherlands	1990-2022	-3.1*									-3.1*
Poland	1990-2002	-3*	2002-2008	-5.6*	2008-2021	-9.5*					-6.3*
Romania	1990-1994	4.6*	1994-2007	-2.5*	2007-2019	-6*					-3*
Spain	1990-1995	3.2*	1995-2004	-3*	2004-2021	-7.6*					-4.6*
UK	1990-2000	-5.3*	2000-2020	-2.5*							-3.4*
Mexico	1990-2020	-3.1*									-3.1*
Argentina	1990-1999	-3.1*	1999-2020	-6.2*							-5.3*
Brazil	1990-2005	-1.2*	2005-2020	-4.2*							-2.7*
Canada	1990-2007	-4.1*	2007-2020	-8.2*							-5.9*
USA	1990-1997	-4.6*	1997-2005	-2*	2005-2020	-7*					-5.1*
Japan	1990-1998	0.8	1998-2013	-3.2*	2013-2021	-6.2*					-3*
Australia	1990-2022	-2.5*									-2.5*

APC: annual percent change. AAPC: average annual percent change.
* Significantly different from zero (*p*<0.05).

Supplementary Table 2. Joinpoint analysis from 1990 to the most available year for females aged 25-49 years, by country and cancer site.

	Years1	APC1	Years2	APC2	Years3	APC3	Years4	APC4	Years5	APC5	AAPC
**All cancers**											
France	1990-2002	0.1	2002-2020	-2.1*							-1.2*
Germany	1990-1993	0.1	1993-2006	-3*	2006-2020	-1.6*					-2*
Italy	1990-2020	-1.7*									-1.7*
Netherlands	1990-2003	-0.6*	2003-2022	-2.8*							-1.9*
Poland	1990-1995	-0.5	1995-2005	-2.2*	2005-2008	-4.5	2008-2021	-2.5*			-2.3*
Romania	1990-1997	1*	1997-2006	-1.4*	2006-2010	-5.2*	2010-2017	-0.4	2017-2019	-7.3*	-1.5*
Spain	1990-1994	0.4	1994-2006	-1.8*	2006-2021	-2.5*					-1.9*
UK	1990-2009	-2.5*	2009-2020	-1*							-1.9*
Canada	1990-2020	-2.2*									-2.2*
USA	1990-2020	-1.7*									-1.7*
Mexico	1990-2012	-1.5*	2012-2020	0.7*							-0.9*
Argentina	1990-2001	-0.8*	2001-2008	-2.1*	2008-2020	0.4*					-0.6*
Brazil	1990-2020	0.2*									0.2*
Japan	1990-2000	-0.7*	2000-2003	-3.9	2003-2021	-1.6*					-1.6*
Australia	1990-2022	-2.1*									-2.1*
**Colorectum**											
France	1990-2020	-0.6*									-0.6*
Germany	1990-2006	-3.5*	2006-2020	0							-1.9*
Italy	1990-2020	-2.3*									-2.3*
Netherlands	1990-2022	-0.4*									-0.4*
Poland	1990-2021	-1*									-1*
Romania	1990-2019	-0.3									-0.3
Spain	1990-2021	-2.1*									-2.1*
UK	1990-1994	-6.2*	1994-2004	-1.9*	2004-2007	5.7	2007-2011	-1.9	2011-2020	3.8*	-0.1
Canada	1990-2005	-2.1*	2005-2020	0.6							-0.7*
USA	1990-2004	-0.3	2004-2020	0.8*							0.3*
Mexico	1990-2020	2.1*									2.1*
Argentina	1990-2020	0.9*									0.9*
Brazil	1990-1998	7.1*	1998-2020	1.3*							2.8*
Japan	1990-2021	-1.5*									-1.5*
Australia	1990-2010	-1.5*	2010-2022	1.3*							-0.5
**Pancreas**											
France	1990-2020	1*									1*
Germany	1990-2020	-0.4*									-0.4*
Italy	1990-2020	0.4									0.4
Netherlands	1990-2022	-0.7									-0.7

	Years1	APC1	Years2	APC2	Years3	APC3	Years4	APC4	Years5	APC5	AAPC
Poland	1990-2021	-1.5*									-1.5*
Romania	1990-2019	-0.2									-0.2
Spain	1990-2011	1.5*	2011-2021	-2.6*							0.1
UK	1990-2020	-0.3									-0.3
Canada	1990-2020	-0.9*									-0.9*
USA	1990-2007	0.5	2007-2020	-1*							-0.1
Mexico	1998-2020	-0.7*									-0.7*
Argentina	1997-2020	-0.1									-0.1
Brazil	1996-2020	1.5*									1.5*
Japan	1990-2021	-0.3									-0.3
Australia	1990-2022	-0.2									-0.2
**Lung**											
France	1990-2004	7.6*	2004-2014	-2.7*	2014-2020	-6.7*					1.1*
Germany	1990-2005	2.3*	2005-2020	-4.5*							-1.2*
Italy	1990-1995	4.5*	1995-1998	-3.6	1998-2002	5.2	2002-2012	-1.7*	2012-2020	-3.9*	-0.6
Netherlands	1990-2004	4.4*	2004-2022	-6.1*							-1.6*
Poland	1990-1996	4.3*	1996-2005	-1	2005-2021	-6.3*					-2.8*
Romania	1990-2019	0.5*									0.5*
Spain	1990-2004	7.1*	2004-2015	-2.8*	2015-2021	-8.3*					0.4
UK	1990-1999	-0.9	1999-2020	-2.7*							-2.2*
Canada	1990-2005	-0.6	2005-2020	-8.7*							-4.7*
USA	1990-1998	-2.4*	1998-2005	0.9*	2005-2011	-4.6*	2011-2020	-8.5*			-4*
Mexico	1990-2020	-2*									-2*
Argentina	1990-2006	1*	2006-2009	-11.6	2009-2020	-1.2*					-1.1
Brazil	1990-2008	1.9*	2008-2020	-2.6*							0.1
Japan	1990-1996	2.2*	1996-2021	-3.4*							-2.4*
Australia	1990-2009	-0.1	2009-2022	-4.9*							-2.1*
**Breast**											
France	1990-1999	0.1	1999-2012	-2.6*	2012-2020	0					-1.1*
Germany	1990-1994	-0.5	1994-2007	-4.4*	2007-2020	-0.3					-2.1*
Italy	1990-2009	-2.5*	2009-2020	-0.6							-1.8*
Netherlands	1990-1999	-1.3	1999-2022	-3.1*							-2.6*
Poland	1990-2007	-2.8*	2007-2021	-0.4							-1.7*
Romania	1990-2001	-0.7*	2001-2011	-3.7*	2011-2017	2.4*	2017-2019	-11*			-1.8*
Spain	1990-2003	-3.5*	2003-2021	-2*							-2.6*
UK	1990-2010	-3.1*	2010-2020	-1.3*							-2.5*
Argentina	1990-2011	-2*	2011-2020	1.3*							-1*
Brazil	1990-2003	-0.1	2003-2020	1.2*							0.6*
Canada	1990-2004	-3.6*	2004-2020	-1.8*							-2.6*
Mexico	1990-1996	2*	1996-2013	-1.2*	2013-2020	1.6*					0.1

	Years1	APC1	Years2	APC2	Years3	APC3	Years4	APC4	Years5	APC5	AAPC
USA	1990-2007	-3.1*	2007-2020	-1.4*							-2.3*
Japan	1990-1999	2.1*	1999-2021	-1.6*							-0.6*
Australia	1990-2022	-2.9*									-2.9*

APC: annual percent change. AAPC: average annual percent change.

* Significantly different from zero (*p*<0.05).

Supplementary Table 3. Age-adjusted incidence rates per 1000,000 males from colorectal, pancreatic, lung cancer and all cancers combined among males aged 25-49 years in major countries worldwide in selected countries worldwide in 2010 and 2017.

	**All cancers**			**Colorectum**			**Pancreas**			**Lung**		
	**ASIR 2010**	**ASIR 2017**	**% change**	**ASIR 2010**	**ASIR 2017**	**% change**	**ASIR 2010**	**ASIR 2017**	**% change**	**ASIR 2010**	**ASIR 2017**	**% change**
France	128.44	121.88	-5.11	10.05	9.52	-5.27	1.76	2.14	21.59	15.12	11.98	-20.77
Germany	114.69	115.84	1.00	11.45	11.71	2.27	2.58	1.81	-29.84	7.74	7.20	-6.98
Netherlands	113.33	115.43	1.85	11.97	12.27	2.51	1.61	1.48	-8.07	7.57	6.08	-19.68
Spain	110.51	100.28	-9.26	11.41	10.06	-11.83	2.61	1.85	-29.12	10.2	7.51	-26.37
UK	98.29	106.84	8.70	9.07	12.23	34.84	1.63	1.73	6.13	5.34	4.51	-15.54
Canada	102.62	105.11	2.43	11.95	14.01	17.24	1.72	1.80	4.65	5.21	3.52	-32.44
USA	127.94	121.21	-5.26	13.01	17.10	31.44	2.01	2.52	25.37	6.21	4.09	-34.14
Japan	80.05	84.39	5.42	13.00	15.96	22.77	1.70	2.22	30.59	6.91	6.95	0.58
Australia	142.11	140.26	-1.30	15.02	16.57	10.32	2.34	2.11	-9.83	5.41	4.49	-17.01

ASIR, age-adjusted incidence rates using the world standard population.

Supplementary Table 4. Age-adjusted incidence rates per 1000,000 females from colorectal, pancreatic, lung cancer and all cancers combined among males aged 25-49 years in major countries worldwide in selected countries worldwide in 2010 and 2017.

	**All cancers**			**Colorectum**			**Pancreas**			**Lung**			**Breast**		
	**ASIR 2010**	**ASIR 2017**	**% change**	**ASIR 2010**	**ASIR 2017**	**% change**	**ASIR 2010**	**ASIR 2017**	**% change**	**ASIR 2010**	**ASIR 2017**	**% change**	**ASIR 2010**	**ASIR 2017**	**% change**
France	216.94	224.07	3.29	8.58	11.65	35.78	1.38	2.74	98.55	9.27	9.47	2.16	98.46	98.00	-0.47
Germany	197.13	204.94	3.96	10.74	10.85	1.02	2.47	1.04	-57.89	8.01	5.53	-30.96	85.96	81.92	-4.70
Netherlands	198.36	215.85	8.82	11.00	12.57	14.27	1.56	1.62	3.85	9.60	7.90	-17.71	90.09	99.41	10.35
Spain	164.48	180.10	9.50	8.94	10.55	18.01	1.18	2.06	74.58	6.32	5.48	-13.29	72.84	81.11	11.35
UK	179.92	192.72	7.11	8.89	12.32	38.58	1.23	1.36	10.57	4.80	4.30	-10.42	80.39	81.66	1.58
Canada	193.03	199.93	3.57	11.00	13.32	21.09	1.36	1.33	-2.21	5.49	3.80	-30.78	67.89	71.10	4.73
USA	212.02	216.74	2.23	12.76	15.26	19.59	1.87	1.96	4.81	6.21	4.12	-33.66	77.62	82.07	5.73
Japan	167.12	198.53	18.79	11.74	13.08	11.41	1.44	1.32	-8.33	5.40	4.67	-13.52	72.80	87.43	20.10
Australia	207.75	223.64	7.65	13.89	16.93	21.89	1.17	2.09	78.63	4.69	4.97	5.97	78.63	82.57	5.01

ASIR, age-adjusted incidence rates using the world standard population.

Supplementary Table 5. Age-standardized (world population) mortality rates per 100,000 males aged 25-49 years for selected cancer sites in major countries worldwide in 2009-2011 and 2019-2021°, with the corresponding percent change between the two calendar periods.

		**Oralcav/Ph**	**Esophagus**	**Stomach**	**Liver**	**Skin**	**Testis**	**Bladder**	**Kidney**	**HL**	**NHL**	**Leukemias**
**France**	ASMR 2009-2011	2.05	1.1	1.54	1.53	1.27	0.48	0.47	1.07	0.36	1.2	1.39
	Average number of deaths 2009-2011	234	126	174	171	140	50	53	121	37	130	149
	ASMR 2019-2021	1.11	0.68	1.2	0.95	1.16	0.38	0.31	0.84	0.16	0.91	0.94
	Average number of deaths 2019-2021	127	79	134	106	127	39	35	95	16	102	99
	% change (95% CI)	-45.9 (-52.5; -39.3)	-38.2 (-44.9; -31.5)	-22.1 (-25.2; -19.0)	-37.9 (-44.2; -31.6)	-8.7 (-9.9; -7.5)	-20.8 (-26.1; -15.5)	-34 (-44.2; -23.8)	-21.5 (-25.2; -17.8)	-55.6 (-77.9; -33.3)	-24.2 (-28.0; -20.4)	-32.4 (-38.0; -26.8)
**Germany**	ASMR 2009-2011	1.76	1.14	1.69	0.65	1.06	0.54	0.29	0.83	0.17	0.92	1.25
	Average number of deaths 2009-2011	323	209	298	113	178	80	54	148	28	154	198
	ASMR 2019-2021	0.96	0.9	1.37	0.71	0.86	0.47	0.22	0.62	0.1	0.66	0.96
	Average number of deaths 2019-2021	133	126	190	98	118	63	31	86	14	91	130
	% change (95% CI)	-45.5 (-51.7; -39.3)	-21.1 (-24.3; -17.9)	-18.9 (-21.1; -16.7)	9.2 (7.3; 11.1)	-18.9 (-9.9; -7.5)	-13.0 (-15.7; -10.3)	-24.1 (-31.1; -17.1)	-25.3 (-29.8; -20.8)	-41.2 (-60.1; -22.3)	-28.3 (-33; -23.6)	-23.2 (-26.4; -20.0)
**Italy**	ASMR 2009-2011	1.21	0.44	1.68	1.1	1.33	0.41	0.31	0.79	0.4	1.4	1.68
	Average number of deaths 2009-2011	149	55	207	135	159	43	38	97	42	163	191
	ASMR 2019-2021	0.9	0.46	1.37	0.91	1.13	0.51	0.23	0.88	0.2	0.97	1.36
	Average number of deaths 2019-2021	103	54	150	101	119	48	27	100	20	99	135
	% change (95% CI)	-25.6 (-29.6; -21.6)	4.5 (3.3; 5.7)	-18.5 (-21; -16)	-17.3 (-20.1; -14.5)	-15.0 (-21.9; -15.9)	24.4 (17.3; 31.5)	-25.8 (-33.9; -17.7)	11.4 (9.1; 13.7)	-50.0 (-67.8; -32.2)	-30.7 (-35.6; -25.8)	-19.0 (-21.6; -16.4)
**Netherlands**	ASMR 2009-2011	0.88	1.4	1.17	0.66	2.29	0.53	0.38	1.11	0.25	1.09	1.19
	Average number of deaths 2009-2011	29	48	39	21	73	15	13	37	7	35	36
	ASMR 2019-2021	0.39	1.15	0.97	0.68	1.16	0.43	0.27	0.7	0.14	1.04	0.96
	Average number of deaths 2019-2021	11	34	28	20	33	12	8	20	4	30	27
	% change (95% CI)	-55.7 (-78.4; -33.0)	-17.9 (-22.2; -13.6)	-17.1 (-21.9; -12.3)	3.0 (1.5; 4.5)	-49.3 (-61.3; -37.3)	-18.9 (-27.2; -10.6)	-28.9 (-44.9; -12.9)	-36.9 (-48.6; -25.2)	-44.0 (-81.7; -6.3)	-4.6 (-5.7; -3.5)	-19.3 (-24.9; -13.7)
**Poland**	ASMR 2009-2011	2.42	0.92	2.92	0.41	1.44	1.05	0.57	1.37	0.54	1.17	1.76
	Average number of deaths 2009-2011	160	60	194	28	97	74	37	91	38	80	120

		Oralcav/Ph	Esophagus	Stomach	Liver	Skin	Testis	Bladder	Kidney	HL	NHL	Leukemias
	ASMR 2019-2021	2.48	0.82	1.89	0.43	0.92	1.23	0.32	0.75	0.29	1.07	1.05
	Average number of deaths 2019-2021	182	60	139	32	68	86	23	56	21	78	74
	% change (95% CI)	2.5 (2.1; 2.9)	-10.9 (-13.0; -8.8)	-35.3 (-39.5; -31.1)	4.9 (3.0; 6.8)	-36.1 (-42.6; -29.6)	17.1 (13.8; 20.4)	-43.9 (-57.0; -30.8)	-45.3 (-54.0; -36.6)	-46.3 (-61.4; -31.2)	-8.5 (-9.9; -7.1)	-40.3 (-46.8; -33.8)
**Romania**	ASMR 2009-2011	6.73	1.42	3.79	0.83	1.25	0.9	0.56	1.15	0.55	1.49	1.91
	Average number of deaths 2009-2011	248	52	143	31	49	36	21	42	21	58	77
	ASMR 2019-2021	4.8	1.19	2.53	0.65	1.18	0.6	0.44	1.05	0.28	1.09	1.32
	Average number of deaths 2019-2021	196	49	100	26	48	22	18	42	11	41	51
	% change (95% CI)	-28.7 (-33.1; -24.3)	-16.2 (-21.2; -11.2)	-33.2 (-40.5; -25.9)	-21.7 (-31.2; -12.2)	-5.6 (-7.1; -4.1)	-33.3 (-48.8; -17.8)	-21.4 (-32.2; -10.6)	-8.7 (-11.4; -6.0)	-49.1 (-82.9; -15.3)	-26.8 (-35.6; -18)	-30.9 (-40.3; -21.5)
**Spain**	ASMR 2009-2011	1.52	1.02	1.67	1.82	0.87	0.31	0.51	0.86	0.39	1.24	1.28
	Average number of deaths 2009-2011	144	97	161	173	85	30	48	83	36	120	121
	ASMR 2019-2021	0.73	0.6	1.32	0.82	0.62	0.24	0.23	0.87	0.16	0.83	0.94
	Average number of deaths 2019-2021	71	60	129	79	57	19	23	86	15	79	83
	% change (95% CI)	-52.0 (-60.3; -43.7)	-41.2 (-49.3; -33.1)	-21.0 (-23.8; -18.2)	-54.9 (-62.9; -46.9)	-28.7 (-34.5; -22.9)	-22.6 (-29.5; -15.7)	-54.9 (-71.2; -38.6)	1.2 (0.8; 1.6)	-59 (-83.9; -34.1)	-33.1 (-38.9; -27.3)	-26.6 (-30.9; -22.3)
**UK**	ASMR 2009-2011	0.92	1.66	1.01	0.87	1.41	0.32	0.31	1	0.33	1.14	1.18
	Average number of deaths 2009-2011	109	197	118	101	162	34	36	118	36	130	130
	ASMR 2019-2021	0.99	1.37	0.99	0.8	0.94	0.21	0.3	0.88	0.13	0.87	1.04
	Average number of deaths 2019-2021	111	155	111	90	107	24	34	100	15	98	116
	% change (95% CI)	7.6 (6.1; 9.1)	-17.5 (-19.8; -15.2)	-2.0 (-2.2; -1.8)	-8.0 (-9.3; -6.7)	-33.3 (-38.2; -28.4)	-34.4 (-45.8; -23.0)	-3.2 (-3.9; -2.5)	-12.0 (-13.8; -10.2)	-60.6 (-81.9; -39.3)	-23.7 (-27.6; -19.8)	-11.9 (-13.7; -10.1)
**Canada**	ASMR 2009-2011	0.7	0.98	0.97	0.77	1.16	0.28	0.32	0.73	0.32	1.36	1.17
	Average number of deaths 2009-2011	48	69	67	53	78	17	22	51	19	91	75
	ASMR 2019-2021	0.77	0.9	0.84	0.72	0.8	0.37	0.2	0.59	0.14	0.81	1.01
	Average number of deaths 2019-2021	50	59	54	47	53	24	13	39	9	53	64
	% change (95% CI)	10.0 (7.0; 13.0)	-8.2 (-9.8; -6.6)	-13.4 (-16.3; -10.5)	-6.5 (-7.9; -5.1)	-31.0 (-38.2; -23.8)	32.1 (18.5; 45.7)	-37.5 (-55.1; -19.9)	-19.2 (-24.4; -14)	-56.3 (-84.3; -28.3)	-40.4 (-49.4; -31.4)	-13.7 (-16.5; -10.9)
**USA**	ASMR 2009-2011	0.8	1.05	1.02	0.96	1.32	0.41	0.31	0.93	0.34	1.32	1.72
	Average number of deaths 2009-2011	458	609	579	554	739	211	180	537	178	731	929

		Oralcav/Ph	Esophagus	Stomach	Liver	Skin	Testis	Bladder	Kidney	HL	NHL	Leukemias
	ASMR 2019-2021	0.64	0.92	0.97	0.79	0.84	0.46	0.23	0.78	0.15	0.91	1.36
	Average number of deaths 2019-2021	348	498	530	431	461	252	122	421	82	494	743
	% change (95% CI)	-20.0 (-21.5; -18.5)	-12.4 (-13.2; -11.6)	-4.9 (-5.2; -4.6)	-17.7 (-19.1; -16.3)	-36.4 (-39.3; -33.5)	12.2 (10.5; 13.9)	-25.8 (-28.4; -23.2)	-16.1 (-17.4; -14.8)	-55.9 (-63.8; -48)	-31.1 (-33.5; -28.7)	-20.9 (-22.2; -19.6)
**Argentina**	ASMR 2009-2011	0.94	0.97	2.34	0.23	0.88	1.57	0.35	1.29	0.38	1.94	1.95
	Average number of deaths 2009-2011	58	58	143	14	56	106	21	78	26	126	127
	ASMR 2019-2021	0.7	0.62	1.93	0.3	0.63	1.69	0.23	1.19	0.27	1.65	1.99
	Average number of deaths 2019-2021	52	45	143	22	48	131	17	87	22	125	153
	% change (95% CI)	-25.5 (-31.6; -19.4)	-36.1 (-45.4; -26.8)	-17.5 (-20.0; -15)	30.4 (14.1; 46.7)	-28.4 (-35.2; -21.6)	7.6 (6.2; 9.0)	-34.3 (-48.3; -20.3)	-7.8 (-9.1; -6.5)	-28.9 (-39.1; -18.7)	-14.9 (-17.1; -12.7)	2.1 (1.6; 2.6)
**Brazil**	ASMR 2009-2011	2.61	2.29	2.69	0.5	0.75	0.44	0.24	0.51	0.24	1.22	1.68
	Average number of deaths 2009-2011	876	760	921	169	262	161	80	175	86	433	601
	ASMR 2019-2021	1.95	1.58	2.21	0.34	0.63	0.62	0.19	0.51	0.25	1.1	1.62
	Average number of deaths 2019-2021	739	596	865	133	248	246	71	196	101	436	640
	% change (95% CI)	-25.3 (-26.8; -23.8)	-31.0 (-33.3; -28.7)	-17.8 (-18.8; -16.8)	-32.0 (-36.3; -27.7)	-16.0 (-17.8; -14.2)	40.9 (35.4; 46.4)	-20.8 (-26.1; -15.5)	0.0 (-0.1; 0.1)	4.2 (3.3; 5.1)	-9.8 (-10.6; -9.0)	-3.6 (-3.8; -3.4)
**Mexico**	ASMR 2009-2011	0.45	0.37	2.3	0.38	0.46	1.18	0.18	0.78	0.39	1.32	2.51
	Average number of deaths 2009-2011	81	64	418	68	83	227	31	134	73	245	476
	ASMR 2019-2021	0.44	0.37	2.33	0.4	0.36	1.84	0.18	1	0.42	1.25	2.79
	Average number of deaths 2019-2021	93	78	500	85	77	413	39	209	91	272	612
	% change (95% CI)	-2.2 (-2.4; -2.0)	0 (-0.2; 0.2)	1.3 (1.1; 1.5)	5.3 (3.9; 6.7)	-21.7 (-26.0; -17.4)	55.9 (49.9; 61.9)	0.0 (-0.3; 0.3)	28.2 (24.1; 32.3)	7.7 (5.9; 9.5)	-5.3 (-5.8; -4.8)	11.2 (10.2; 12.2)
**Japan**	ASMR 2009-2011	0.86	0.64	3.03	1.77	0.15	0.21	0.18	0.45	0.06	0.91	1.47
	Average number of deaths 2009-2011	191	141	668	390	35	45	40	100	13	198	313
	ASMR 2019-2021	0.65	0.4	1.78	0.77	0.16	0.14	0.19	0.35	0.02	0.6	1.11
	Average number of deaths 2019-2021	148	97	401	181	35	28	44	84	4	130	227
	% change (95% CI)	-24.4 (-27.4; -21.4)	-37.5 (-42.4; -32.6)	-41.3 (-44.2; -38.4)	-56.5 (-61.7; -51.3)	6.7 (3.8; 9.6)	-33.3 (-44.4; -22.2)	5.6 (3.5; 7.7)	-22.2 (-25.9; -18.5)	-66.7 (-138.9; 5.5)	-34.1 (-38.4; -29.8)	-24.5 (-26.8; -22.2)
**Australia**	ASMR 2009-2011	1.07	1.02	1.1	1.14	2.51	0.23	0.17	0.85	0.2	0.84	1.19
	Average number of deaths 2009-2011	44	42	45	47	102	9	7	35	8	34	47

		Oralcav/Ph	Esophagus	Stomach	Liver	Skin	Testis	Bladder	Kidney	HL	NHL	Leukemias
	ASMR 2019-2021	0.87	0.79	0.96	0.87	1.31	0.31	0.12	0.61	0.12	0.65	0.96
	Average number of deaths 2019-2021	39	35	43	39	58	14	5	27	5	29	43
	% change (95% CI)	-18.7 (-23.1; -14.3)	-22.5 (-28.3; -16.7)	-12.7 (-15.6; -9.8)	-23.7 (-29.5; -17.9)	-47.8 (-56.5; -39.1)	34.8 (16.5; 53.1)	-29.4 (-52.3; -6.5)	-28.2 (-36.3; -20.1)	-40.0 (-67.5; -12.5)	-22.6 (-28.8; -16.4)	-19.3 (-23.6; -15.0)

° The triennium 2019-2021 was evaluated for Spain Poland, Netherlands, Japan, and Australia while the 2019-2020 for the other countries considered.
ASMR, Age-standardized mortality rates; CI, confidence interval.

Supplementary Table 6. Age-standardized (world population) mortality rates per 100,000 females aged 25-49 years for selected cancer sites in major countries worldwide in 2009-2011 and 2019-2021°, with the corresponding percent change between the two calendar periods.

		Oralcav/Ph	Esophagus	Stomach	Liver	Skin	Uterus	Ovary	Bladder	Kidney	HL	NHL	Leukemias
**France**	ASMR 2009-2011	0.54	0.24	0.88	0.47	1.05	2.55	1.36	0.19	0.41	0.26	0.58	1.05
	Average number of deaths 2009-2011	62	28	100	53	117	295	154	22	47	26	65	114
	ASMR 2019-2021	0.31	0.21	0.83	0.45	0.72	2.01	1.07	0.16	0.34	0.06	0.46	0.83
	Average number of deaths 2019-2021	36	25	95	53	83	233	122	19	40	7	52	90
	% change (95% CI)	-42.6 (-54.9; -30.3)	-12.5 (-17; -8.0)	-5.7 (-6.6; -4.8)	-4.3 (-5.1; -3.5)	-31.0 (-37.5; -25.3)	-21.2 (-23.5; -18.9)	-21.3 (-24.5; -18.1)	-15.8 (-22.2; -9.4)	-17.0 (-21.5; -12.7)	-77.0 (-130.9; -22.9)	-21.0 (-25.7; -15.7)	-21.0 (-24.6; -17.4)
**Germany**	ASMR 2009-2011	0.50	0.20	1.32	0.33	0.95	2.37	1.79	0.23	0.35	0.08	0.51	1.02
	Average number of deaths 2009-2011	85	35	218	54	153	393	303	38	58	12	80	155
	ASMR 2019-2021	0.33	0.20	1.18	0.42	0.57	2.19	1.52	0.20	0.29	0.03	0.45	0.75
	Average number of deaths 2019-2021	45	27	161	57	78	298	205	27	41	5	61	99
	% change (95% CI)	-34.0 (-41.1; -26.9)	0.0 (-0.4; 0.4)	-10.6 (-11.9; -9.3)	27.3 (19.8; 34.8)	-40.0 (-47.9; -32.1)	-7.6 (-8.3; -6.9)	-15.1 (-16.8; -13.4)	-13.0 (-17.2; -8.8)	-17.0 (-21.4; -12.8)	-63.0 (-106.9; -18.1)	-12.0 (-14.1; -9.5)	-26.5 (-30.7; -22.3)
**Italy**	ASMR 2009-2011	0.46	0.08	1.47	0.31	1.12	1.80	1.78	0.12	0.33	0.29	0.81	1.29
	Average number of deaths 2009-2011	56	11	180	37	135	225	222	15	42	31	94	151
	ASMR 2019-2021	0.41	0.11	1.10	0.27	0.87	1.91	1.71	0.15	0.38	0.15	0.60	0.80
	Average number of deaths 2019-2021	47	12	123	30	93	208	195	18	41	16	60	85
	% change (95% CI)	-10.9 (-13.5; -8.3)	37.5 (8.1; 66.9)	-25.2 (-28.8; -21.6)	-13.0 (-17.1; -8.7)	-22.0 (-26.4; -18.2)	6.1 (5.2; 7.0)	-3.9 (-4.3; -3.5)	25.0 (10.6; 39.4)	15.2 (10.7; 19.7)	-48.0 (-69.7; -26.9)	-26.0 (-31.7; -20.1)	-38.0 (-44.4; -31.6)
**Netherlands**	ASMR 2009-2011	0.22	0.26	1.07	0.31	1.93	1.74	1.71	0.37	0.36	0.10	0.73	1.15
	Average number of deaths 2009-2011	7	9	35	10	60	55	55	12	12	3	22	35
	ASMR 2019-2021	0.21	0.31	0.93	0.48	0.93	2.12	1.47	0.25	0.33	0.06	0.41	0.72
	Average number of deaths 2019-2021	6	9	27	14	26	61	42	7	10	2	11	20
	% change (95% CI)	-4.5 (-7.2; -1.8)	19.2 (7.1; 31.3)	-13.1 (-16.7; -9.5)	54.8 (24.7; 84.9)	-52.0 (-65.2; -38.4)	21.8 (16.8; 26.8)	-14.0 (-17; -11)	-32.4 (-49.5; -15.3)	-8.3 (-12.1; -4.5)	-40.0 (-98.8; 18.8)	-44.0 (-62.2; -25.4)	-37.4 (-48.9; -25.9)
**Poland**	ASMR 2009-2011	0.51	0.17	1.59	0.12	1.01	4.88	3.5	0.16	0.51	0.41	0.71	1.15
	Average number of deaths 2009-2011	34	12	106	8	68	324	232	11	34	28	48	78
	ASMR 2019-2021	0.51	0.17	1.35	0.23	0.70	2.97	2.54	0.23	0.3	0.19	0.56	0.86

		Oralcav/Ph	Esophagus	Stomach	Liver	Skin	Uterus	Ovary	Bladder	Kidney	HL	NHL	Leukemias
	Average number of deaths 2019-2021	37	12	98	16	51	218	183	17	22	13	40	62
	% change (95% CI)	0.0 (-0.3; 0.3)	0.0 (-0.5; 0.5)	-15.1 (-17.4; -12.8)	91.7 (49.5; 133.9)	-31.0 (-37.2; -24.2)	-39.1 (-43.0; -35.2)	-27.4 (-30.4; -24.4)	43.8 (21.1; 66.5)	-41.0 (-54.6; -27.8)	-54.0 (-73.3; -34.1)	-21.0 (-26; -16.2)	-25.2 (-29.9; -20.5)
**Romania**	ASMR 2009-2011	0.69	0.19	1.80	0.35	1.01	11.21	2.84	0.16	0.54	0.25	1.16	1.25
	Average number of deaths 2009-2011	27	7	71	13	39	433	109	6	20	10	45	49
	ASMR 2019-2021	0.59	0.21	1.57	0.28	0.60	7.33	2.22	0.33	0.37	0.25	1.13	1.02
	Average number of deaths 2019-2021	22	8	58	11	22	275	84	11	14	8	41	36
	% change (95% CI)	-14.5 (-21.3; -7.7)	10.5 (0.5; 20.5)	-12.8 (-16.4; -9.2)	-20.0 (-34.6; -5.4)	-41.0 (-59.5; -21.7)	-34.6 (-39.1; -30.1)	-21.8 (-26.8; -16.8)	106.0 (5.6; 207)	-32.0 (-50.1; -12.9)	0 (0.9; -0.9)	-2.6 (-3.2; -2)	-18.4 (-24.8; -12.0)
**Spain**	ASMR 2009-2011	0.43	0.15	1.35	0.38	0.75	2.25	1.84	0.19	0.34	0.22	0.59	1.02
	Average number of deaths 2009-2011	40	13	127	36	71	213	174	18	32	20	55	93
	ASMR 2019-2021	0.25	0.08	1.11	0.34	0.52	1.86	1.45	0.13	0.35	0.13	0.49	0.81
	Average number of deaths 2019-2021	24	8	105	33	50	175	142	13	33	10	43	68
	% change (95% CI)	-41.9 (-54.4; -29.4)	-46.7 (-73.2; -20.2)	-17.8 (-20.3; -15.3)	-11.0 (-13.1; -7.9)	-31.0 (-36.7; -24.7)	-17.3 (-19.2; -15.4)	-21.2 (-23.8; -18.6)	-31.6 (-45.9; -17.3)	2.9 (1.6; 4.2)	-41.0 (-57.9; -23.9)	-17.0 (-20.6; -13.2)	-20.6 (-24.3; -16.9)
**UK**	ASMR 2009-2011	0.41	0.40	0.69	0.45	1.05	2.7	1.78	0.31	0.44	0.18	0.74	0.87
	Average number of deaths 2009-2011	50	47	81	53	122	308	212	38	53	19	85	99
	ASMR 2019-2021	0.42	0.30	0.76	0.61	0.76	2.56	1.60	0.21	0.37	0.07	0.49	0.71
	Average number of deaths 2019-2021	49	35	87	71	87	291	184	24	43	8	56	79
	% change (95% CI)	2.4 (1.6; 3.2)	-25 (-32.3; -17.7)	10.1 (8.0; 12.2)	35.6 (26.7; 44.5)	-28.0 (-32.7; -22.5)	-5.2 (-5.6; -4.8)	-10.1 (-11.2; -9)	-32.3 (-43.1; -21.5)	-16.0 (-20.1; -11.7)	-61.0 (-98.2; -24)	-34.0 (-41.8; -25.8)	-18.4 (-21.9; -14.9)
**Canada**	ASMR 2009-2011	0.27	0.16	0.8	0.48	0.76	2.27	1.75	0.21	0.28	0.2	0.71	1.08
	Average number of deaths 2009-2011	18	11	53	32	50	152	119	14	19	12	47	69
	ASMR 2019-2021	0.36	0.19	0.62	0.51	0.50	2.39	1.51	0.14	0.28	0.04	0.48	0.68
	Average number of deaths 2019-2021	23	12	40	33	33	156	98	9	19	3	32	44
	% change (95% CI)	33.3 (18.7; 47.9)	18.8 (6.6; 31.0)	-22.5 (-28.3; -16.7)	6.3 (4.0; 8.6)	-34.0 (-43.7; -24.7)	5.3 (4.4; 6.2)	-13.7 (-15.9; -11.5)	-33.3 (-50.5; -16.1)	0.0 (-0.5; 0.5)	-80.0 (-164.9; 4.9)	-32.0 (-41.9; -22.9)	-37.0 (-46.1; -27.9)
**USA**	ASMR 2009-2011	0.29	0.21	0.79	0.40	0.86	2.92	1.74	0.17	0.38	0.22	0.74	1.30
	Average number of deaths 2009-2011	165	119	439	225	472	1624	985	99	213	117	405	702
	ASMR	0.27	0.18	0.84	0.44	0.58	3.02	1.43	0.14	0.31	0.09	0.49	1.00

		Oralcav/Ph	Esophagus	Stomach	Liver	Skin	Uterus	Ovary	Bladder	Kidney	HL	NHL	Leukemias
	2019-2021												
	Average number of deaths 2019-2021	148	99	456	239	316	1657	775	74	167	47	266	544
	% change (95% CI)	-6.9 (-7.8; -6.0)	-14.3 (-16.2; -12.4)	6.3 (5.7; 6.9)	10.0 (8.5; 11.5)	-33.0 (-35.2; -30.0)	3.4 (3.2; 3.6)	-17.8 (-18.9; -16.7)	-17.6 (-20.6; -14.6)	-18.0 (-20.8; -16)	-59.0 (-72.8; -45.4)	-34.0 (-37.0; -30.6)	-23.1 (-24.7; -21.5)
**Argentina**	ASMR 2009-2011	0.34	0.38	1.55	0.14	0.61	9.38	2.29	0.12	0.60	0.19	1.00	1.64
	Average number of deaths 2009-2011	23	25	103	9	41	638	150	8	39	13	69	112
	ASMR 2019-2021	0.24	0.34	1.47	0.16	0.56	11.73	2.17	0.17	0.66	0.19	0.75	1.46
	Average number of deaths 2019-2021	19	26	115	13	44	930	167	14	51	15	60	115
	% change (95% CI)	-29.4 (-41.2; -17.6)	-10.5 (-14; -7.0)	-5.2 (-5.9; -4.5)	14.3 (4.3; 24.3)	-8.2 (-10.3; -6.1)	25.1 (23.4; 26.8)	-5.2 (-5.8; -4.6)	41.7 (19.6; 63.8)	10.0 (6.8; 13.2)	0.0 (-0.5; 0.5)	-25.0 (-30.6; -19.4)	-11.0 (-12.7; -9.3)
**Brazil**	ASMR 2009-2011	0.48	0.40	1.85	0.25	0.52	6.19	1.59	0.12	0.30	0.21	0.80	1.46
	Average number of deaths 2009-2011	174	143	675	90	188	2264	573	43	109	77	294	543
	ASMR 2019-2021	0.45	0.28	1.74	0.24	0.42	6.88	1.60	0.11	0.34	0.16	0.75	1.32
	Average number of deaths 2019-2021	183	113	710	98	171	2836	642	44	139	66	305	539
	% change (95% CI)	-6.3 (-6.9; -5.7)	-30.0 (-35.0; -25.0)	-5.9 (-6.2; -5.6)	-4.0 (-4.7; -3.3)	-19.0 (-21.4; -17)	11.1 (10.7; 11.5)	0.6 (0.5; 0.7)	-8.3 (-10.1; -6.5)	13.3 (10.8; 15.8)	-24.0 (-27.3; -20.3)	-6.3 (-6.9; -5.7)	-9.6 (-10.3; -8.9)
**Mexico**	ASMR 2009-2011	0.24	0.11	2.21	0.38	0.41	6.44	2.29	0.11	0.39	0.25	0.85	2.22
	Average number of deaths 2009-2011	48	23	438	72	83	1270	447	22	75	52	171	455
	ASMR 2019-2021	0.24	0.10	2.24	0.38	0.31	6.33	2.69	0.08	0.50	0.23	0.87	2.35
	Average number of deaths 2019-2021	58	24	528	88	73	1504	630	20	116	55	204	555
	% change (95% CI)	0.0 (-0.2; 0.2)	-9.1 (-11.3; -6.9)	1.4 (1.2; 1.6)	0.0 (-0.2; 0.2)	-24.0 (-30; -18.8)	-1.7 (-1.7; -1.7)	17.5 (16.1; 18.9)	-27.3 (-35.4; -19.2)	28.2 (22.5; 33.9)	-8.0 (-9.7; -6.3)	2.4 (2.0; 2.8)	5.9 (5.4; 6.4)
**Japan**	ASMR 2009-2011	0.42	0.18	2.74	0.39	0.21	3.51	2.33	0.08	0.21	0.03	0.55	1.05
	Average number of deaths 2009-2011	91	38	599	84	46	770	507	17	44	5	115	219
	ASMR 2019-2021	0.35	0.23	1.78	0.30	0.15	3.43	2.00	0.10	0.15	0.01	0.36	0.73
	Average number of deaths 2019-2021	75	54	380	65	32	755	450	23	33	1	76	146
	% change (95% CI)	-16.7 (-19.6; -13.8)	27.8 (19.5; 36.1)	-35.0 (-37.5; -32.5)	-23.0 (-26.8; -19.4)	-29.0 (-37.7; -19.5)	-2.3 (-2.4; -2.2)	-14.2 (-15.2; -13.2)	25.0 (16.4; 33.6)	-29.0 (-37.7; -19.5)	-67.0 (-141.6; 8.2)	-35.0 (-39.7; -29.3)	-30.5 (-34.4; -26.6)
**Australia**	ASMR 2009-2011	0.29	0.19	0.79	0.44	1.64	1.67	1.27	0.14	0.38	0.14	0.53	0.87
	Average number of deaths 2009-2011	12	8	33	18	67	69	52	6	16	6	22	35
	ASMR	0.30	0.18	0.73	0.61	0.96	1.91	1.13	0.08	0.25	0.06	0.29	0.61

		Oralcav/Ph	Esophagus	Stomach	Liver	Skin	Uterus	Ovary	Bladder	Kidney	HL	NHL	Leukemias
	2019-2021												
	Average number of deaths 2019-2021	14	8	33	28	43	87	51	4	11	3	13	27
	% change (95% CI)	3.4 (1.1; 5.7)	-5.3 (-8.3; -2.3)	-7.6 (-9.5; -5.7)	38.6 (23.9; 53.3)	-42.0 (-50.4; -32.6)	14.4 (11.5; 17.3)	-11.0 (-13.2; -8.8)	-42.9 (-74; -11.8)	-34.0 (-50; -18.4)	-57.0 (-106.6; -7.6)	-45.0 (-65.1; -25.5)	-29.9 (-38.9; -20.9)

° The triennium 2019-2021 was evaluated for Spain Poland, Netherlands, Japan, and Australia while the 2019-2020 for the other countries considered.

ASMR, Age-standardized mortality rates; CI, confidence interval.
